# Structural Characterization of Peptides From Huangjiu and Their Regulation of Hepatic Steatosis and Gut Microbiota Dysbiosis in Hyperlipidemia Mice

**DOI:** 10.3389/fphar.2021.689092

**Published:** 2021-06-15

**Authors:** Ying Shi, Ruixue Feng, Jieqi Mao, Shuangping Liu, Zhilei Zhou, Zhongwei Ji, Shuguang Chen, Jian Mao

**Affiliations:** ^1^ National Engineering Laboratory for Cereal Fermentation Technology, Jiangnan University, Wuxi, China; ^2^ School of Food Science and Technology, Jiangnan University, Wuxi, China; ^3^ Jiangnan University (Shaoxing) Industrial Technology Research Institute, Shaoxing, China; ^4^ College of Agriculture and Environmental Sciences, University of California, Davis, CA, United States; ^5^ National Engineering Research Center of Chinese Rice Wine, Zhejiang Guyuelongshan Shaoxing Wine CO., Ltd, Shaoxing, China; ^6^ Department of General Surgery, Peking Union Medical College Hospital, Chinese Academy of Medical Sciences and Peking Union Medical College (CAMS and PUMC), Beijing, China

**Keywords:** hyperlipidemia, hepatic steatosis, gut dysbiosis, microbe interactions, dietary interventions, food-derived peptides

## Abstract

Hyperlipidemia is a chronic disorder that is difficult to cure and usually treated with long-term lipid-reducing drugs. Recent trends have led to the use of diet therapies or food-derived strategies in the treatment of such long-term diseases. The Chinese rice wine (huangjiu) contains a wide range of bioactive peptides that are produced during the multi-species fermentation process. To clarify the regulation effects of lipid metabolism and gut microbiota by huangjiu bioactive peptides, three huangjiu peptides were isolated, purified and characterized by hyper-filtration, macroporous resin, gel filtration separation and structural identification. Meanwhile, a mouse model of high-fat diet-induced hyperlipidemia was established to study the effects of huangjiu peptides on serum biomarker, hepatic metabolism and gut microbiota dysbiosis. Experimental results showed that huangjiu peptides T1 and T2 (HpT1, HpT2) treatment alleviated the increase in serum total cholesterol, triglyceride, low-density lipoprotein cholesterol levels and aberrant hepatic lipid accumulation in the high-fat diet-induced hyperlipidemia mice. Furthermore, HpT2 and HpT1 restored the *α*-diversity and structure of gut microbial community after hyperlipidemia-induced microbiota disturbance compared with simvastatin and HpT3. The administration of HpT2 and HpT1 regulated the microbiota-mediated gut ecology through alterations of characteristic taxa including *Lactobacillus*, *Ileibacterium*, *Faecalibaculum* and *Alloprevotella* by linear discriminant analysis effect size analysis. Collectively, our results offer new insights into the abilities of food-derived peptides on alleviation of high-fat diet-induced hyperlipidemia, hepatic steatosis and gut dysbiosis in mice.

## Introduction

Chinese rice wine (huangjiu) is famous for its low alcohol content (12–18%vol), amber color, pleasant flavor, mild taste and abundant functional components, and it has the history of brewing and consumption for more than 5,000 years. The abundant peptides, amino acids, polysaccharides and phenols in huangjiu are generated during the complex brewing process (Ren et al., 2019). Particularly, the yeast, fungi and lactic acid bacteria can transform the proteins in glutinous rice and wheat into numerous diverse and unique polypeptides during the 30–90-days fermentation. Ultra-pure liquid chromatography-electrospray ion trap-tandem mass spectrometry studies have tentatively identified more than 500 peptides in huangjiu, including 43 potential bioactive peptides and three sensory-active peptides (Han and Xu, 2011). However, the functions of huangjiu peptides have rarely been studied.

Hyperlipidemia is a type of metabolic dysfunction characterized by abnormal serum lipid levels, such as increased triglyceride (TG), total cholesterol (TC) and low-density lipoprotein cholesterol (LDL-C) levels and relatively lower high-density lipoprotein cholesterol (HDL-C) levels. Also, hyperlipidemia is a major risk factor in the development of hepatic steatosis, liver damage, type 2 diabetes, hypertension, atherosclerosis and other metabolic diseases (Zhong et al., 2018). Although several drugs, including nicotinic acid, bile acid sequestrants, fibrates and statins, have been widely used to treat hyperlipidemia, these agents have toxic effects and limitation in long-term treatment (Wu et al., 2009). An earlier meta-analysis that focused on the safety and potential adverse effect data from 35 trials of statin therapy indicated possible risks of interaction with cholesterol, stoke, or high risk of cardiovascular (Ramkumar et al., 2016).

Gut dysbiosis is a common side effect of chronic diseases such as hyperlipidemia, obesity and diabetes. Huangjiu peptides, which are food-derived, have an advantage over hypolipidemic drugs in the reversal of high-fat diet-induced gut dysbiosis. The long-term intake and lower side effects of huangjiu peptides could be advantageous in the treatment of gut microbiota dysbiosis-induced metabolic diseases (Li et al., 2018). Muscle pain is a common side effect of simvastatin use, and the long-term use of this drug for hyperlipidemia could potentially cause gut microbiota disorders or additional adverse effects. Antihypertensive drugs are also associated with adverse effects such as peripheral neuropathy and other central nervous system-related effects, anti-diuretic hormone secretion and collagen-related effects (Merel and Paauw, 2017). These increased incidences of known risk side effects vary widely among patients and are difficult to prognosticate. Therefore, the development and application of effective daily dietary strategies for hyperlipidemia and other chronic conditions are especially crucial.

The results of recent studies have proven the abilities of several bioactive peptides on regulating signaling pathways relevant to lipid metabolism. For example, three peptides (IAVPGEVA, IAVPTGVA and LPYP) isolated from soy glycinin were shown to modulate cholesterol metabolism in HepG2 cells through the LDLR-SREBP2 pathway (Lammi et al., 2015). Furthermore, a novel mitochondrial-derived peptide was shown to regulate metabolic homeostasis *via* AMPK activation in diet-induced obesity mice (Lee et al., 2015). Val-Ser-Glu-Glu, a peptide isolated from duck eggs, inhibited abnormal lipid metabolism in a Wnt/*β*-catenin signaling pathway-dependent mechanism in an ovariectomized rat model, and Val-Ser-Glu-Glu treatment regulated abundances of Firmicutes, Veillonellaceae, Prevotellaceae and six genera. (Guo et al., 2019). These food-derived bioactive peptides exhibited cholesterol-lowering activities, the relationships between the peptide type, dose and effect remain uncertain. The potential effectiveness of novel peptides and their beneficial activation of daily intake remain to be studied. Importantly, although huangjiu contains great amount of nitrogen-containing compounds which largely in the forms of peptides and amino acids, many of them have not been characterized and their potential bioactivities should be further studied (Zhou et al., 2021).

In this study, three novel peptides were isolated, purified and characterized from huangjiu to detect and evaluate their function in high-fat diet-induced hyperlipidemia mice. The hyperlipidemia model was confirmed by determining the serum levels of lipid-related enzymes and hepatic levels of TC and TG. The different huangjiu peptides (HpT1, HpT2 and HpT3) were measured and compared to investigate the improvement of aberrant lipid metabolism and modulation of liver injury. Furthermore, their impact on the restoration of intestinal microbiota were evaluated by LEfSe and Tax4Fun analyses in the dysbiotic state. Our findings provide a new insight that administration of different huangjiu peptides in hyperlipidemia mice exhibited divergent effects in regulation of the lipid metabolism and gut microbial community.

## Materials and Methods

### Extraction, Purification and Preparation of Huangjiu Peptides

The Shaoxing huangjiu samples were collected from Guyuelongshan Shaoxing Wine Co., ltd. in China with the aging time more than 5 years. The sample of Shaoxing huangjiu (volume: 1 L) was vacuum-filtered through a 0.22-*μ*m-pore filter attached to a circulating vacuum pump to isolate the relatively lower-molecular weight fractions. Subsequently, hyper-filtration was applied to obtain components with molecular weights <3 kDa. The hyper-filtration apparatus was pre-treated using a 0.1 mol L^−1^ NaOH solution, and the extracted sample was preserved for further purification after rotary evaporation. Type DA201-C macroporous resin was selected for purification, and an optimum adsorption time of 3 h was determined by calculating the adsorption rate. The eluate from the resin was collected and subjected to rotary evaporation and lyophilization to obtain huangjiu peptides. These crude peptides were designated the CP component. A gel chromatography column (Sephadex G-15) was used to further separate and purify the CP components according to a modified version of previous method (Mäkinen, 2014). A 16 mg mL^−1^ solution of the CP component was prepared with distilled water. After filtration through a 0.22 *µ*m microporous membrane, the sample was applied to the gel filtration separation chromatography system. Distilled water (pH 7.0) was chosen as the mobile phase with a flow rate of 3 ml min^−1^, and the eluent was automatically collected by the instrument. Two main peaks were separated, and the eluent from the same separation peak was collected and freeze-dyed, and they were named as G1 and G2, respectively.

### Structure Identification of Huangjiu Peptides

The amino acid sequence of the purified huangjiu component G1 was identified using an electrospray ion trap mass spectrometer (ESI-IT-MS, Thermo). The sample was reductively alkylated and separated by reverse-phase chromatography with mobile phases A (0.1% formic acid, 5% acetonitrile) and B (0.1% formic acid, 75% acetonitrile). The elution conditions are shown in Supplementary Table S1, and MS was conducted according to previously reported methods (Thaysen-Andersen et al., 2011). To identify the corresponding amino acid sequences, the original mass spectrometry files were retrieved using the Maxquant database. Three huangjiu peptides were identified and designated as huangjiu peptides T1, T2 and T3 (HpT1, HpT2 and HpT3, respectively). The isolated peptide products were processed by rotary evaporation and freeze-drying for subsequent animal gavage.

### Animals and Experimental Design

Four-week-old male C57BL/6 mice (specific pathogen-free) were obtained from Slack Experimental Animal Co., Ltd. (Shanghai, China). Throughout the experiments, mice were caged in groups of two or three and housed under strictly controlled light conditions (12-h/12-h light/dark cycle), and distilled water was provided *ad libitum*. The experimental procedures and number of animals were approved by the Ethics Committee of Jiangnan University, China. The experimental procedures were carried out in accordance with the American Veterinary Medical Association (AVMA) Guidelines and all efforts were made to minimize suffering.

Each mouse was allocated to one of six groups (control group, Con; high-fat diet group, Hfd; simvastatin treatment group, SiT; huangjiu peptide T1 group, HpT1; huangjiu peptide T2 group, HpT2; huangjiu peptide T3 group, HpT3; *n* = 7–10 in each group and listed in Supplementary Table S2) and acclimatized for 1 week prior to the experiments. All groups except the control group were fed with a high-fat diet (TP28640, Paigen diet series) for 8 weeks. The control group was provided with a normal diet (TP28462, Paigen diet series) for 8 weeks. All treatments were delivered by once-daily gavage for 8 weeks during the diet-feeding period. Mice in the three peptide groups received 0.3 g kg^−1^ body weight of a peptide solution, respectively, in HpT1, HpT2 or HpT3 groups. The dose of simvastatin treatment group (6 mg kg^−1^) was calculated according to the drug instruction of hyperlipidemia therapy, while the dose of huangjiu peptide groups was determined according to the dietary supplement of food intake.

### Collection of Blood, Tissue and Fecal Samples and Histologic Analyses of Liver Tissue

Blood was collected from the mice in all groups (*n* = 7–10) by exsanguination and stored for 3 h at room temperature. Serum was collected by centrifugation (4°C, 4,000 g for 15 min) and stored at −80°C until further analysis. Pieces of liver tissue were collected, weighted and diluted (1:10 v/v) in phosphate-buffered saline (PBS, pH 7.4) on ice, followed by homogenization with a Scientz-50 tissue mill (Lanzhi, Ningbo, China) and centrifugation at 13,000 g for 10 min to obtain the supernatants. Fecal pellets were collected from mice in each group (*n* = 7–10) at week eight for the microbiome analyses. The mice were placed in a sterile room and the feces of single mouse were collected separately. Subsequently, the fresh fecal samples were transferred into sterile precooled tubes and stored at −80°C within 30 min of collection.

For the histological analyses, harvested liver tissues were flushed with PBS, fixed in a 4% paraformaldehyde solution overnight at room temperature and transferred into 75% ethanol. The dehydration and section of each tissue slide was conducted by Automatic Dehydration Machine (LEICA, LeicaASP300S, Germany) and Semi-Automatic Slicer (LEICA, Leica RM2245, Germany). After staining fixed tissue sections with hematoxylin and eosin (H&E) by Automatic Dyeing Machine (LEICA, Leica TS5015, Germany), the tissue slides were observed under a microscope (Olympus BX45, Japan) for histopathological examination (Olympus DP72 Image Analysis System, Japan). Pathological scores were assigned by averaging six fields per sample, as described previously (Yeh and Brunt, 2014).

### Measurement of Biochemical Indexes, Cytokines and Hormones in Serum and Liver Samples

Biochemical indexes in serum were measured using an automatic biochemical analyzer (BS-480; Mindray Biomedical Electronics Co., Ltd.) including TC, TG, HDL-C, LDL-C, alanine transaminase (ALT) and aspartate aminotransferase (AST). The concentrations of TC, TG and malondialdehyde (MDA) in the supernatants of homogenized liver samples were measured using assay kits (Jiancheng Bioengineering Institute, Nanjing, China).

### Deoxyribonucleic Acid Extraction, Sequencing, and Analyses

Total genomic DNA was extracted from the fecal samples using the Fast DNA Spin Kit (MP Biomedical, United States). Subsequently, 16S rRNA gene was amplified by a polymerase chain reaction (PCR) that targeted the V3–V4 region, as described previously (Shi et al., 2018a). The annealing temperature and extend time are 63.8°C for 30 s and 42.3°C for 30 s for V3 and V4, respectively, and the PCR condition was 94°C, 5 min; 94°C, 30 s, annealing temperature for 30 s and 72°C for extend time, repeat for 30 cycles; 72°C, 7 min. The resulting library was subjected to paired-end sequencing on an Illumina Nova sequencing platform. The sequencing data were analyzed using the QIIME (Quantitative Insights Into Microbial Ecology) pipeline. QIIME scripts (assign_taxonomy.py, pick_otus.py, make_otu_table.py) were used to cluster the sequences into operational taxonomic units (OTUs) with ≥97% identity after de-multiplexing and quality control (split_libraries.py, QualityFilter20. pl). The OTU sequences were aligned after removing the chimeric sequences (seqs_chimeras_filtered; USEARCH6.1).

The *α*-diversity and *β*-diversity were estimated according to the ecological diversity and similarity between the microbial communities as determined through the UniFrac analysis and unweighted principal coordinate analysis, respectively. The heatmaps of different groups were drafted in R studio using the *vegan* package. The linear discriminate analysis effect size (LEfSe) algorithm was applied (Shi et al., 2018b), and the non-parametric Kruskal–Wallis test was used to identify significantly different taxa in abundances followed by linear discriminate analysis to determine the effect size. Tax4Fun was applied to predict the functional profiles of gut microbiota based on KEGG Orthology (KO) terms, which linked 16S rRNA gene sequences with the functional annotation of sequenced prokaryotic genomes (Asshauer et al., 2015; Weng et al., 2016).

### Statistical Analysis

Data were represented as means ± SEM. The statistical analysis of data was subjected to a one-way ANOVA, followed by Dunnett’s multiple comparisons test in GraphPad Prism seven for Windows. Comparisons between high-fat diet-treated mice and control mice were made using unpaired two-tailed *t* tests. A *p* value <0.05 was considered to indicate statistical significance.

## Results

### Isolation and Identification of Novel Huangjiu Peptides

To separate the various and complex components in huangjiu, a multi-step peptide purification process was conducted using the following steps including suction filtration, ultrafiltration, macroporous resin purification and gel chromatography. The elution peaks of the huangjiu crude peptides are displayed in Supplementary Figure S1, which showed that component G1 dominated the spectrum. Hence, the amino acid composition of G1 was further evaluated to determine whether hydrophobicity, molecular weight, chain length and other properties might be relevant to peptide bioactivity. As shown in Table 1, glutamate (24.41 ± 0.21 g/100 g) was the most abundant amino acid in component G1, followed by phenylalanine, leucine and glycine. Hydrophobic amino acids accounted for 32.63% of the total amino acids. Moreover, the huangjiu G1 peptide components were subjected to ESI-IT-MS for identification and purification. The specific amino acid sequences and compositions of the three huangjiu peptides were detected and are listed in Supplementary Figure S2, and novelty of these peptides was verified in the NCBI database (https://blast.ncbi.nlm.nih.gov/Blast.cgi). The sequences of HpT1, HpT2 and HpT3 were Tyr-Val-Lys-Val (YVKV), Leu-Phe-Trp (LFW) and Phe-Leu-Phe (FLF), respectively, and these had corresponding molecular weights of 507.30, 464.24, and 425.23 Da.

**TABLE 1:** Amino acid composition of fraction G1.

Amino acids	Content (g·100 g^−1^)	Amino acids	Content (g·100 g^−1^)
Aspartic acid	2.55 ± 0.05	Valine	2.15 ± 0.05
Glutamic acid	24.41 ± 0.21	Methionine	0.32 ± 0.02
Serine	1.72 ± 0.02	Phenylalanine	3.25 ± 0.05
Histidine	0.82 ± 0.02	Isoleucine	2.25 ± 0.05
Glycine	2.90 ± 0.01	Leucine	2.94 ± 0.04
Threonine	2.05 ± 0.05	Lysine	0.51 ± 0.01
Arginine	0.72 ± 0.02	Proline	1.84 ± 0.04
Tyrosine	1.33 ± 0.03	Hydrophobic amino acids	32.63
Alanine	1.01 ± 0.01	Alkaline amino acids	3.92
Cysteine	2.09 ± 0.09	Essential amino acids	28.46

### Huangjiu Peptides T1 and T2 Alleviate Hepatic Lipid Accumulation and Aberrant Lipid Metabolism in Mice Fed a High-Fat Diet

**FIGURE 1:**
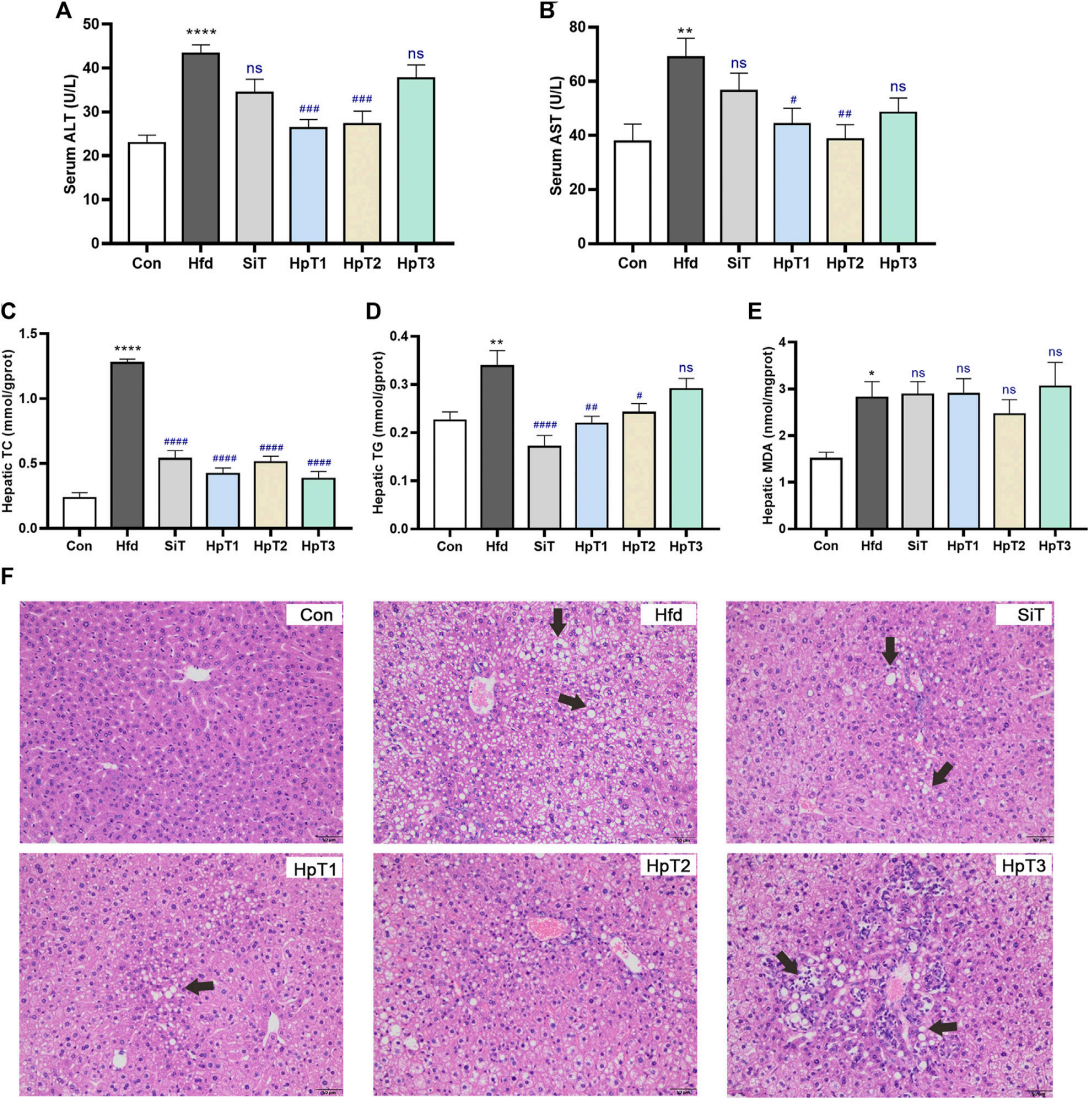
Huangjiu peptide T1 and T2 treatment alleviated aberrant lipid metabolism in the liver induced by high-fat diet. Mice were fed with high-fat diet for 8 weeks except control (Con) group were treated with normal diet; Mice of the high-fat diet group (Hfd) were treated with saline and fed with high-fat diet for 8 weeks; the simvastatin treatment group (SiT) were treated with simvastatin (6 mg kg^−1^); and huangjiu peptide T1 group (HpT1), huangjiu peptide T2 group (HpT2), huangjiu peptide T3 group (HpT3) were administrated with huangjiu peptide T1, T2 or T3 (0.3 g kg^−1^) by gavage, respectively. These group names were used throughout this research paper. Levels of **(A)** ALT and **(B)** AST in serum were determined by biochemical kits, and these samples were obtained from Con, Hfd, SiT, HpT1, HpT2, HpT3 mice groups. Levels of **(C)** TC, **(D)** TG and **(E)** MDA in liver were measured thought kits and calculated according to hepatic protein content. **(F)** Representative liver tissue sections were stained with H&E to indicate lipid contents and bars = 50 *μ*m. Data were presented as mean ± SEM of six to nine mice per group. Unpaired two-tailed *t* tests were used between Con and Hfd groups, and the black * was labelled on column with *p < 0.05, **p < 0.01, ****p < 0.0001. One-way ANOVA analyses were applied followed by Dunnett’s multiple comparisons test, and the significant *p* value of each group was compared with Hfd group. The blue # label indicated #*p* < 0.05, ##*p* < 0.01, ###*p* < 0.001, ####*p* < 0.0001, and ns means no significant differences.

To evaluate the effects of specific huangjiu peptides on serum and liver profiles of hepatic biomarkers in mice after 8 weeks, we measured the serum levels of metabolizing enzymes associated with liver injury and hepatic levels of TC and TG (Figure 1). The serum ALT and AST levels reflected a severe state of liver injury (*p* < 0.0001 in Figure 1A and *p* < 0.01 in Figure 1B) in high-fat diet (Hfd) group compared to the control (Con) group. Notably, administration with HpT1 and HpT2 remarkably suppressed the increases in these liver biomarkers more efficiently than simvastatin treatment (SiT) group (*p* < 0.001 in Figure 1A and *p* < 0.05, *p* < 0.01, respectively, in Figure 1B). Consistent with the serum profile, our analysis of the hepatic levels of TC, TG and antioxidative markers such as MDA indicated that a high-fat diet induced liver injury (*p* < 0.0001, *p* < 0.01 and *p* < 0.05, respectively, in Figures 1C–E). Especially, HpT1 and HpT2 reversed the increased trend of hepatic TC and TG levels induced by the high-fat diet (Figures 1C,D), suggesting that both peptides are hepato-protective. However, the anti-oxidative effects of simvastatin and the three peptides were not obvious from the analysis of hepatic MDA levels (Figure 1E).

H&E staining of the liver sections (Figure 1F) revealed striking hepatic steatosis in mice fed a high-fat diet. In Hfd group, the disordered arrangement of hepatocytes with moderate or severe steatosis was observed, together with the mild focal inflammation and infiltration occasionally in cells. The bullous or slight follicular steatosis was eliminated, indicating that the liver steatosis was obviously alleviated by administration of drug. The accumulation of larger fat particles and abnormal infiltration of inflammatory cells were inhibited by HpT1 and HpT2. However, HpT3 did not appear to promote the reversal of liver pathology. Additionally, on the basis of the liver injury and steatosis assessment scores in Supplementary Tables S3,S4, the extent of hepatic steatosis was significantly decreased in SiT, HpT1 and HpT2 groups (*p* < 0.05). The analysis of serum and hepatic levels of lipid metabolism biomarkers confirmed that HpT1 and HpT2 alleviated the severe hepatic steatosis resulting from a high-fat diet. The HpT1, HpT2 and HpT3 groups were further compared to reveal the effects of the peptides on the gut microbiota after hyperlipidemia-induced gut dysbiosis and to clarify discrepancies in their effects on lipid modulation.

### Huangjiu Peptides T1 and T2 Modulate Serum Biomarkers of Hyperlipidemia Profile in Mice Fed a High-Fat Diet

**FIGURE 2:**
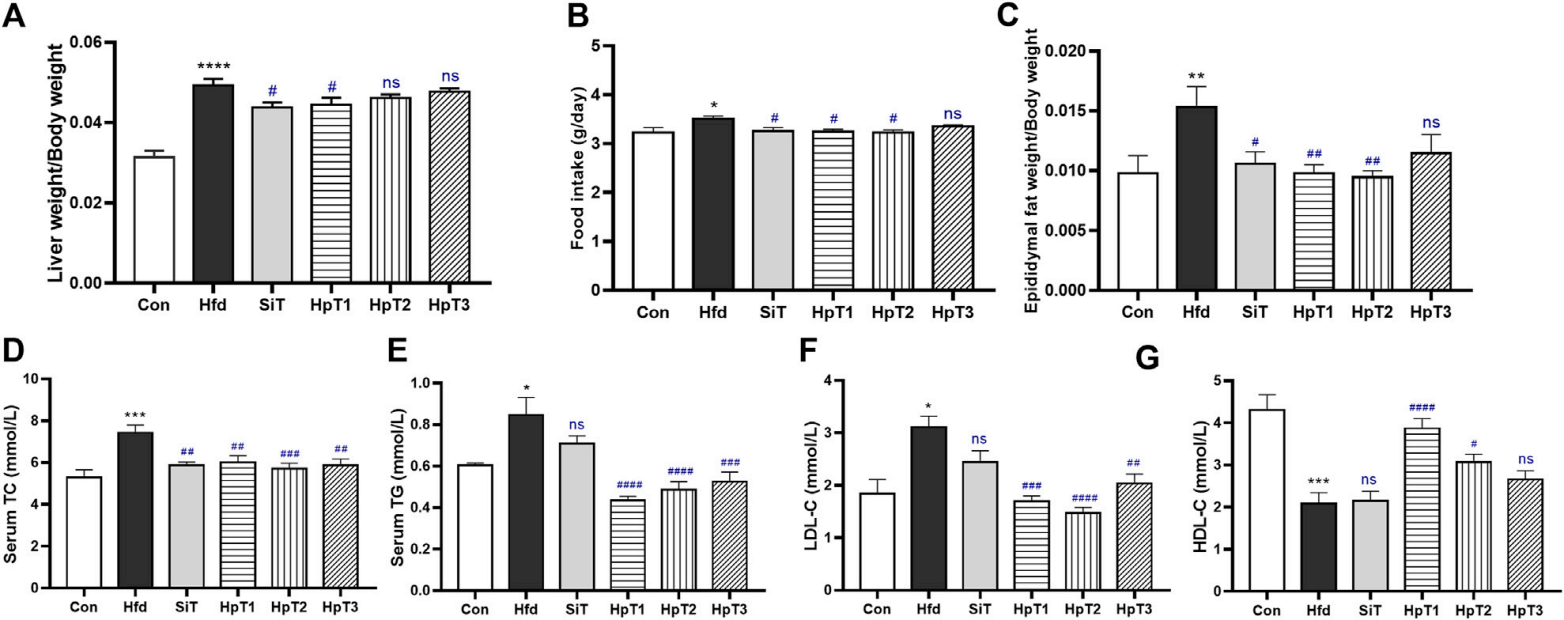
Huangjiu peptides T1 and T2 reduced epididymal fat weight gain and decreased serum TC and LDL-C in high-fat induced mice. **(A)** Liver index and **(B)** food intake were examined among mice groups. **(C)** Level of epididymal fat was measured. **(D)** TC, **(E)** TG **(F)** LDL-C and **(G)** HDL-C of serum samples were assayed by biochemical kits. Data were presented as mean ± SEM of six to nine mice per group. Unpaired two-tailed *t* tests were used between Con and Hfd groups, and the black * was labelled on column with *p < 0.05, **p < 0.01, ***p < 0.001, ****p < 0.0001. One-way ANOVA analyses were applied followed by Dunnett’s multiple comparisons test, and the significant *p* value of each group was compared with Hfd group. The blue # label indicated #*p* < 0.05, ##*p* < 0.01, ###*p* < 0.001, ####*p* < 0.0001, and ns means no significant differences.

After 8 weeks, mice in the Hfd group exhibited a remarkable increase (*p* < 0.0001) in the liver weight index, compared to the Con group (Figure 2A). Mice in the SiT group showed a significantly reduced food intake (*p* < 0.05) relative to the Hfd group (Figure 2B), indicating that dietary restriction may be responsible for the decreases in serum total cholesterol, triglyceride, low-density lipoprotein cholesterol levels. The high-fat diet induced epididymal fat weight gain (*p* < 0.01, Figure 2C), which was alleviated significantly in the SiT, HpT1 and HpT2 groups (*p* < 0.05, *p* < 0.01 and *p* < 0.01, respectively, Figure 2C). The serum levels of TC, TG and LDL-C were significantly increased in the Hfd group (*p* < 0.001, *p* < 0.05, *p* < 0.05, respectively, Figures 2D–F). And three huangjiu peptides groups were more effective than SiT group in recovery of TG and LDL-C levels. In addition, HpT1 and HpT2 treatment promoted a greater increase in the serum HDL-C concentration than simvastatin (*p* < 0.0001 and *p* < 0.05, Figure 2G). These results demonstrate that the high-fat diet disrupted lipid metabolism in mice, while three huangjiu peptides exhibited divergently in reduction of diet-induced lipid accumulation in the serum. Notably, HpT1 and HpT2 led to similar or greater reductions in the TC, TG and LDL-C concentrations, compared to simvastatin.

### Huangjiu Peptides T1 and T2 Restored the Gut Microbial Community and Key Bacterial Taxa After High-Fat Diet-Induced Dysbiosis

**FIGURE 3:**
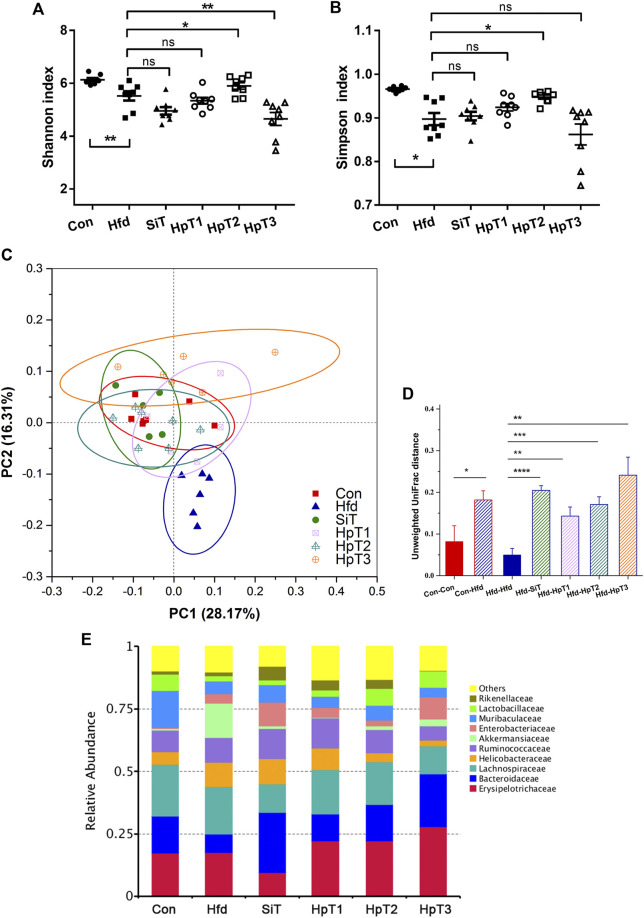
Microbial *α*, *β*-diversity and relative abundance in fecal samples indicated a restorative effect of huangjiu peptides following high fat diet-induced gut dysbiosis. **(A)** *α*-diversity as measured using Shannon index and **(B)** Simpson index from different groups. Data were presented as mean ± SEM of six to nine mice per group. Two-tailed *t*-test was used to determine statistical significance, *p < 0.05, **p < 0.01, ns means no significant differences. **(C)** Principal coordinates analysis (PCoA) clarified differences in microbial community structure between different groups. The first principal component (PC1) and second principal component (PC2) explained 28.17 and 16.31% of the variance in the unweighted UniFrac metrics, respectively. Each point represents the fecal microbiome in a single sample. **(D)** Pairwise distance based on unweighted Unifrac distance measurements and Euclidean distance calculation of microbiota in fecal samples from different mice groups. Data were presented as mean ± SEM of the distances. Unpaired two-tailed *t* tests were used to determine statistical significance between groups, *p < 0.05, **p < 0.01, ***p < 0.001, ****p < 0.0001. **(E)** Alterations of top 10 microbial community at a family level in different groups.

The high-fat diet-induced colonic microbiome disruption was evaluated using 16S rRNA sequencing and diversity analyses. An 8-weeks high-fat diet greatly reduced the bacterial *α*-diversity in the gut, as indicated by the Shannon index and Simpson index (*p* < 0.01 in Figure 3A and *p* < 0.05 in Figure 3B). In all three huangjiu peptide treatment groups, we observed a trend toward an enhanced level of *α*-diversity relative to the normal condition. Particularly, treatment with HpT2 led to obvious improvements in the Shannon and Simpson indexes (Figures 3A,B; *p* < 0.05). The analysis of *β*-diversity based on a principal coordinate analysis (PCoA) revealed that hyperlipidemia altered the composition of the fecal microbiota, which had a considerably separation in microbial community with distance clustering to the Con group (Figures 3C–E). The microbial compositions in the huangjiu peptide-treated mice, particularly those in HpT1 and HpT2 groups, were clustered more closely to the Con group than that to the SiT group, reflecting that huangjiu peptides were more effective than simvastatin for reversing gut microbiota dysbiosis in mice fed with a high-fat diet.

**FIGURE 4:**
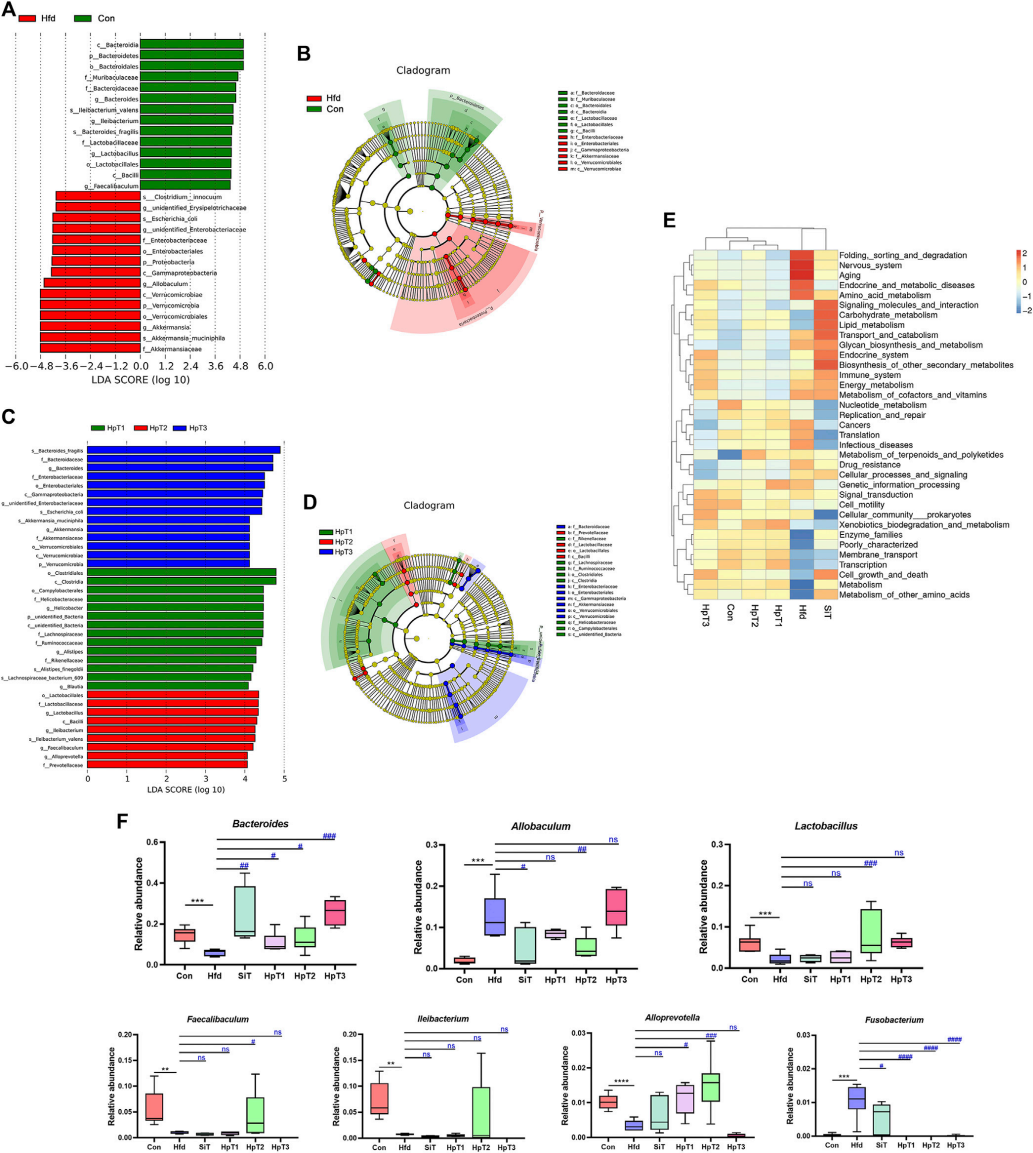
Comparison of differential microbial abundance, characteristic taxa and predicted functions from different mice groups. **(A)** Histogram of taxa with differential abundance in Con and Hfd groups using computed LDA scores. The LDA scores represent the degree of difference abundance in characteristic microbial communities between Con and Hfd groups. **(B)** Circular cladogram of statistically and biologically differences in fecal samples between Con and Hfd groups. Each of circle diameter was proportional to the abundance of taxon. Green = taxon significantly enriched in Con, red = taxon significantly enriched in Hfd and yellow = non-significant. **(C)** Histogram of taxa with differential abundance among HpT1, HpT2 and HpT3 groups using computed LDA scores. The LDA scores represent the degree of difference abundance in characteristic microbial communities among HpT1, HpT2 and HpT3 groups. **(D)** Circular cladogram of statistically and biologically differences in fecal samples among HpT1, HpT2 and HpT3 groups. **(E)** The heatmap of functional comparison and clustering based on Tax4Fun at level 2 among mice groups. The heat map was formed based on top 35 abundant taxa features of samples according to functional annotations and abundance information in the database, and clustered from the level of functional differences. **(F)** Box charts represented relative abundance of *Bacteroides*, *Allobaculum*, *Lactobacillus*, *Faecalibaculum*, *Ileibacterium*, *Alloprevotella*, and *Fusobacterium* in fecal samples from different groups of mice. Boxes represent the interquartile range (IQR), outliers (crosses) (>1.5∗IQR), range (whiskers) and median (horizontal line within the box).

According to the LEfSe analysis based on differentially microbial communities (Figures 4A,B), particular taxa including Verrucomicrobiales, Enterobacteriaceae, *Clostridum* and *Allobaculum* were over-represented in feces from the Hfd group relative to the Con group after consumption of a high-fat diet for 8 weeks. Importantly, administration with HpT2 particularly enhanced the abundances of *Faecalibaculum*, *Ileibacterium*, *Alloprevotella* and *Lactobacillus* to the levels similar to those in the Con group (Figures 4C,D,F). whereas taxa including *Bacteroides*, *Alloprevotella, Lachnospiraceae*, *Alistipes* and *Rikenellaceae* were restored in the HpT1 group. These taxa characteristic clarified that HpT2 and HpT1 improved the abundances of the key taxa while HpT3 had no such effect.

Meanwhile, we generated a taxonomic heatmap clustered by significant taxa based on Bray–Curtis dissimilarity distance, the abundances of *Bacteroides*, *Allobaculum*, *Lactobacillus*, *Alloprevotella* and *Fusobacterium* in the HpT1 and HpT2 groups were restored to the levels in Con group (Supplementary Figure S3), which was in accordance with the LEfSe analysis, indicating that treatment of huangjiu peptides T1, T2 were able to obviously improve the specific gut microbial communities. Taken together, simvastatin did not obviously restore the gut dysbiosis, as shown in a cluster distance analysis (Figure 3C and Supplementary Figure S3), suggesting that drug administration was unable to restore a dysbiotic microbial community to normal state. These data indicated that HpT1 and HpT2 reversed the gut microbiota dysbiosis induced by hyperlipidemia more effectively compared with SiT and HpT3.

### Effects of Huangjiu Peptides on Predicted Microbiota Functions in Hyperlipidemia Mice

To further evaluate interactions on microbiota and metabolites after the huangjiu peptides treatment, microbiota functional profiles were inferred from the 16S rRNA sequencing data. A Tax4Fun analysis was performed to investigate and predict the functional capabilities of the microbial communities with annotations in samples from all groups. The levels of nervous system, aging, infectious diseases were increased and those of enzyme families, membrane transport, cell growth and death were decreased in the Hfd group relative to the Con group (Figure 4E). The functional performances of the HpT2 and HpT1 groups were clustered closer to the Con group, and levels including aging, amino acid metabolism, immune system, replication and repair, translation, and cellular processes and signaling in HpT2 and HpT1 groups were similar with those in the Con group. These results implied that the ability of HpT2 and HpT1 to restore the gut microbiota was crucial to the recovery from hyperlipidemia. Additionally, the curative effect might be related to the immune system or cellular signaling metabolism.

## Discussion

In this study, we purified and identified three novel small peptides from the traditional Chinese fermented alcoholic beverage, huangjiu. To investigate the potential regulatory effects of these peptides on lipid profiles, a mouse model of hyperlipidemia was established by a high-fat diet for 8 weeks, which is commonly used (Baumer et al., 2017). Treatment with simvastatin had a significant effect on the observed dyslipidemia but did not appear to alleviate dysbiosis in the gut microbiota. HpT1 and HpT2 each alleviated hyperlipidemia, mitigated abnormal lipid metabolism and decreased hepatic lipid accumulation. Furthermore, HpT2 restored the gut microbial community after high-fat diet-induced dysbiosis. These results suggest that the potential effects of these peptides on signal transduction and metabolism and their potential clinical use as lipid-lowering agents for hyperlipidemia treatment.

Mice fed with HpT1 or HpT2 exhibited remarkably lower levels of hepatic steatosis and injury than HpT3 groups. Simvastatin treatment group showed obvious effects in some biomarkers including liver weight/body weight serum TC, hepatic TC, hepatic TG and liver steatosis, while had no significant enhancement of high-fat diet-induced gut dysbiosis. In addition, a mouse group of drug simvastatin alleviated the liver injury and steatosis indicated by biochemical and histologic analyses, however, the dysbiosis induced by high-fat diet remained severe and even had slight aggravation. These results supported that peptides T1 and T2 partially compensated the negative consequences of drug. Importantly, the restoration effects of liver injury by bioactive compounds might be associated with gut microbiota, which plays important roles in the development of hyperlipidemia or alcoholic liver disease (Szabo, 2015; Lundgaard et al., 2018).

Food-derived bioactive peptides have varied structures and biological activities. Previous study clarified the relationships between peptides structure and function activity, and demonstrate that the amphipathic conformation of the peptide promotes bacterial cell lysis. And the structure- function relationship of bioactive peptides should be further analyzed based on series of specific experiments. The potentially functional food ingredients could be used for the treatment and prevention of chronic diseases. More importantly, food protein-derived bioactive peptides were proven to exert anti-inflammatory activity by regulating inflammatory cytokines including TNF-α, IL-6, IL-1β and IL-8 *via* the NF-*κ*B or STAT pathway (Tabas and Glass, 2013; Majumder et al., 2016). Although huangjiu peptides are potentially functional peptides derived from fermented food proteins, their safety intake has not been studied clearly. An enzymatic or modifying approach to peptide formation could yield potentially allergenic sequences (Liu L. et al., 2020). Therefore, further evaluation of safety and modulating mechanism of these bioactive peptides, including regulation of metabolome, gut barrier and related immunity pathway, are needed before these peptides can be applied in the clinic.

HpT1 and HpT2 had stronger positive effects than simvastatin on the restoration of gut microbiota after high-fat diet-induced dysbiosis. The relatively stable and dynamic homeostasis of the gut microbiome must be sustained, especially during a long-term treatment of chronic diseases (Jimenez-Giron et al., 2015; Zhernakova et al., 2016; Barroso et al., 2017). In our study, an 8-weeks exposure to a high-fat diet led to remarkable disruption of the intestinal microbial structure, and we observed an obvious divergence in the restoration of specific bacterial genera in the treatment groups. For instance, relative abundance of *Bacteroides* was decreased by high-fat diet, restored by HpT2 and HpT1 relative to the control group. These data are consistent with the finding of a previous study in which the abundance of *Bacteroides* was negatively correlated with obesity and the energy intake (Lee et al., 2019). In another study, restoration of the gut microbiota structure was crucial during treatment of type 2 diabetes and hyperlipidemia with a traditional Chinese herbal formula (Tong et al., 2018). Those findings indicate that changes in specific microbial taxa should be of concern in the chronic gut and liver disease. Only HpT2 enhanced the abundances of *Faecalibaculum* and *Ileibacterium* to the levels in the Con group. In a previous study, andrographolide was used to regulate non-alcoholic fatty liver disease and was shown to decrease the relative abundance of *Bacteroides* while increasing the abundances of *Faecalibaculum* and *Akkermansia* (Shi et al., 2020). Importantly, *Faecalibaculum*, *Bacteroides* and *Akkermansia* appeared to be closely related to the recovery of non-alcoholic fatty liver disease and alteration of gut microbiota in hyperlipidemia (Ma et al., 2019; He et al., 2020). Furthermore, a decrease in the relative abundance of *Ileibacterium* was observed in the context of a metabolism disorder induced by chronic alcohol consumption (Liu Y. et al., 2020). Similarly, the specific restoration of *Ileibacterium* by HpT2 might represent a fundamental step in the mitigation of hyperlipidemia in our case.

An altered gut microbiota contributes significantly to metabolic disorder-related diseases, including dysfunctional glucose and lipid metabolism. The huangjiu peptides could modulate the microbial community through exerting anti-hyperlipidemia activity. The underlying mechanisms of peptides effects in gut microbiota dysbiosis and lipid metabolic disorders have been investigated in recent studies. Supplementation with *Ganoderma lucidum*-derived polysaccharide peptides was shown to modulate the relative abundances of functionally relevant microbial phylotypes, the expression of genes encoding components of hepatic metabolism and the production of total bile acids (Lv et al., 2019). Furthermore, microbiota-dependent alterations could influence lipoprotein metabolism and bile acid signaling *via* bile acid receptors, including farnesoid-X-receptor and Takeda G protein-coupled receptor 5 (Yu et al., 2019). Another study of interactions between a hormone peptide and microbial communities revealed that the microbiota contributed to short-chain fatty acid signaling and affected glucagon-like peptide-1/2 secretion by enteroendocrine L-cells, and thus helped to sustain metabolic homeostasis (Wu et al., 2013; Pathak et al., 2018). Although the regulation of lipids and lipoproteins by the intestinal microbiota has not been widely investigated, evidence suggests that the microbiota modulates the enteroendocrine ecology of the gut and the secretion of certain intestinal peptides. Therefore, our results suggest an urgent need to investigate the interactions between the gut microbiota and metabolic peptides in the regulation of hepatic metabolism.

Our results and hypothesis have prompted us to focus on the metabolome and the correlations between the microbial community and metabolites at the microbiota level in our future research. Although the determination of changes in the gut microbiota can reveal several lines of evidence, further combinations of metabolomics and correlation analyses should be evaluated to reveal the mechanism underlying the restorative effects of huangjiu peptides at the metabolic level (Fang et al., 2019; Huang et al., 2019; Liu et al., 2019). Moreover, by comparing three novel huangjiu peptides and their effects in mice with high-fat diet-induced hyperlipidemia, the differences between the peptides can be explored in the context of applications for specific and targeted diseases (Wu et al., 2017; Lv et al., 2019; Rizo et al., 2020). Meanwhile, the substantial results from our mechanistic analysis of gut dysbiosis revealed discrepant functions of the small huangjiu peptides in the restoration of the gut microbiota. These results provide a new sight that functional compounds in huangjiu could affect alteration of the gut microbiota and liver injury profoundly through gut-liver axis.

## Conclusion

In conclusion, we isolated three small active peptides from huangjiu and revealed that HpT1 and HpT2 could efficiently reduce hepatic steatosis. We observed a significant alleviation of abnormal lipid metabolism and modulation of serum and hepatic biomarker levels by HpT1 and HpT2. Furthermore, we confirmed that both peptides helped to restore the dysbiotic microbial community by regulating specific gut taxa. Compared with simvastatin, HpT1 and HpT2 rather than HpT3 enhanced the intestinal ecology in this chronic disease model. These peptides treatments suggest the potential of a dietary strategy for mediating abnormal lipid accumulation and hyperlipidemia. However, a thorough analysis of underlying metabolic and immune pathway mechanisms is necessary to confirm the beneficial outcomes of these peptides.

## Data Availability

The datasets presented in this study can be found in online repositories. The names of the repository/repositories and accession number(s) can be found in the article/Supplementary Material.
