# Supplementary material for: Structural Characterization of Peptides From Huangjiu and Their Regulation of Hepatic Steatosis and Gut Microbiota Dysbiosis in Hyperlipidemia Mice
Source: Front Pharmacol. 2021 Jun 15;12:689092. doi: 10.3389/fphar.2021.689092 (PMC8243288; doi:10.3389/fphar.2021.689092)
Supplement: Supplementary file 1 [file DataSheet1.docx]

Supplementary Material

Structural characterization of peptides from huangjiu and their regulation of hepatic steatosis and gut microbiota dysbiosis in hyperlipidemia mice

**Ying Shi, Ruixue Feng, Jieqi Mao, Shuangping Liu, Zhilei Zhou, Zhongwei Ji, Shuguang Chen*, Jian Mao****

**Correspondence:** Jian Mao: maojian@jiangnan.edu.cn; Shuguang Chen: cshuguang6@sina.com

# Supplementary Figures and Tables

## Supplementary Figures

**
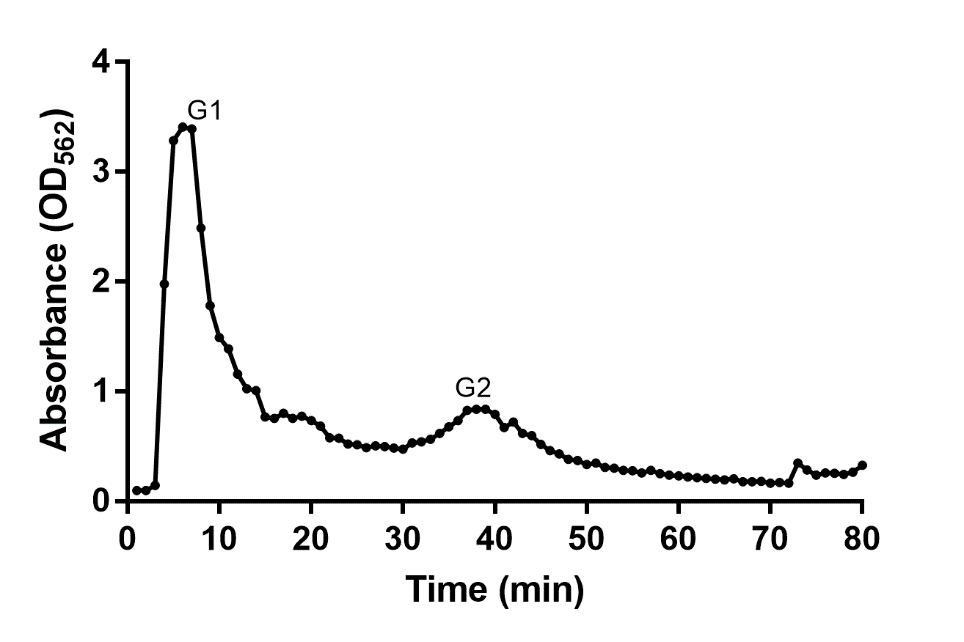
**

**Supplementary Figure 1.** Chromatograms (Sephadex G-15 column) of huangjiu crude peptides. G1 and G2 were represented as two main peaks of absorbance at OD 562.

A


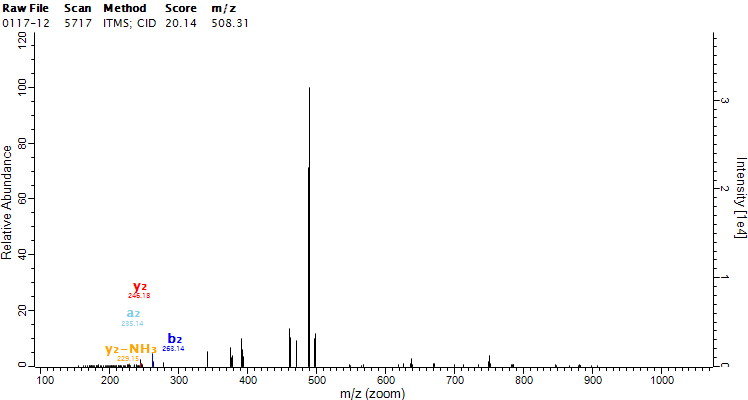


B


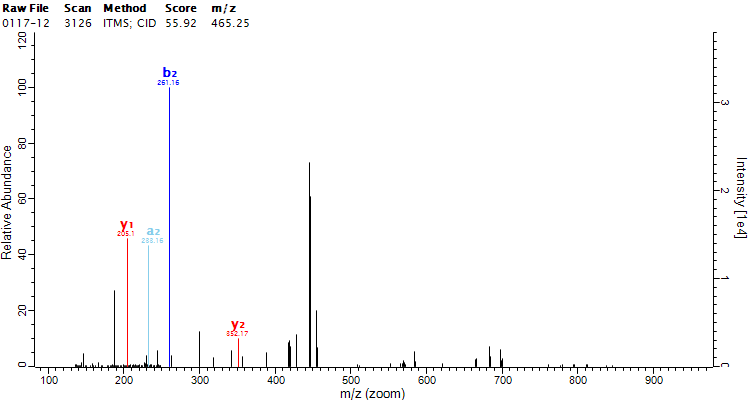


C


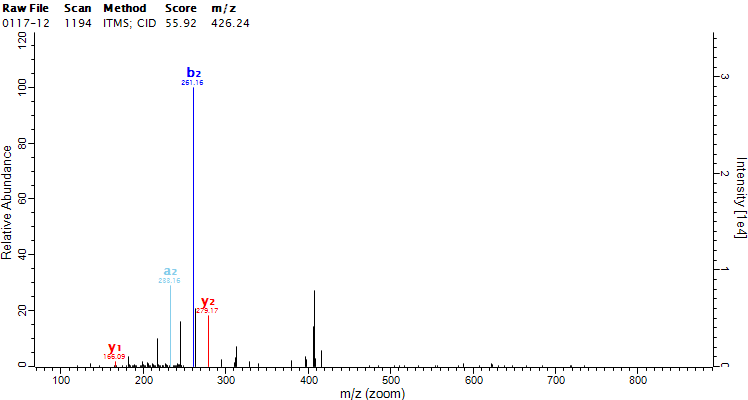


**Supplementary Figure 2.** The mass spectrum (MS) profiles of the huangjiu peptides T1 (A), T2 (B) and T3 (C) analyzed by Maxquant database.


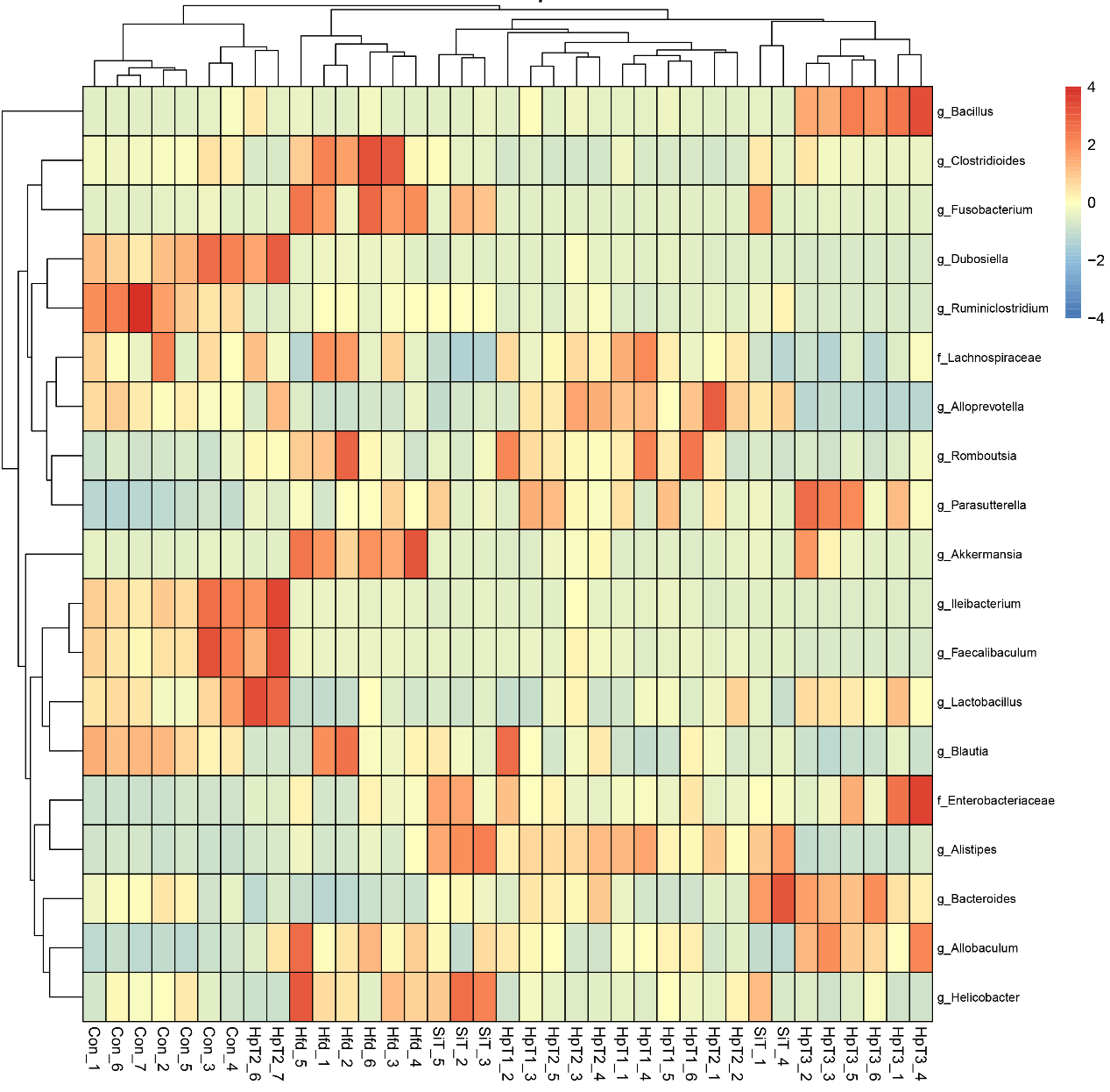


**Supplementary Figure 3. Heatmap of top 19 differential abundance taxa from mice groups based on the genus level.** The clustering of taxonomic heatmap was calculated using Bray-Curtis dissimilarity index distance, combined with Average (unweighted pair-group method with arithmetic means) of statistically significant OTUs. Red and blue represent high and low relative abundance, respectively, and n=5-7 mice per group.

## Supplementary Tables

**Supplementary Table 1**

**Gradient elution conditions of high performance liquid chromatography**

| Time (min) | Flow rate (mL min^-1^) | Mobile phase A (%) | Mobile phase B (%) |
| --- | --- | --- | --- |
| 0 | 0.2 | 94 | 6 |
| 10 | 0.2 | 91 | 9 |
| 25 | 0.2 | 85 | 15 |
| 65 | 0.2 | 70 | 30 |
| 80 | 0.2 | 55 | 45 |
| 85 | 0.2 | 5 | 95 |

**Supplementary Table 2**

**Animal experimental design**

| Groups | Feed | Treatment (8 weeks) |
| --- | --- | --- |
| Control group (Con) | Normal diet | 0.9% saline |
| High-fat diet group (Hfd) | High-fat diet | 0.9% saline |
| Simvastatin treatment group (SiT) | High-fat diet | 6 mg·kg^-1^·simvastatin in 0.9% saline solution |
| Huangjiu peptide T1 group (HpT1) | High-fat diet | 0.3 g·kg^-1^·huangjiu peptide YVKV (T1) in 0.9% saline solution |
| Huangjiu peptide T2 group (HpT2) | High-fat diet | 0.3 g·kg^-1^·huangjiu peptide LFW (T2) in 0.9% saline solution |
| Huangjiu peptide T3 group (HpT3) | High-fat diet | 0.3 g·kg^-1^·huangjiu peptide FLF (T3) in 0.9% saline solution |

**Formula and energy composition of different diet**

|  | High-fat diet | | Normal diet | |
| --- | --- | --- | --- | --- |
|  | gm% | kcal% | gm% | kcal% |
| Protein | 24.0 | 20 | 19.2 | 20 |
| Carbohydrate | 41.0 | 35 | 67.3 | 70 |
| Fat | 24.0 | 45 | 4.3 | 10 |
| Total |  | 100 |  | 100 |
| kcal/gm | 4.73 |  | 3.85 |  |

**Supplementary Table 3**

**The overall liver injury assessment of tissue slides among different mice groups**

| Groups | Number of samples | Mean values | SD values | *P* values (Compared with Con group) | *P* values (Compared with Hfd group) |
| --- | --- | --- | --- | --- | --- |
| Con | 4 | 0.00 | 0.00 |  |  |
| Hfd | 4 | 3.67 | 0.58 | 0.000058 |  |
| SiT | 4 | 2.33 | 0.58 | - | 0.074 |
| HpT1 | 4 | 2.67 | 1.15 | - | 0.172 |
| HpT2 | 4 | 2.00 | 0.00 | - | 0.029 |
| HpT3 | 4 | 3.00 | 1.00 | - | 0.325 |

**Supplementary Table 4**

**The liver steatosis assessment of tissue slides among different mice groups**

| Groups | Number of samples | Mean values | SD values | *P* values (Compared with Con group) | *P* values (Compared with Hfd group) |
| --- | --- | --- | --- | --- | --- |
| Con | 4 | 0.00 | 0.00 |  |  |
| Hfd | 4 | 3.33 | 0.58 | 0.000014 |  |
| SiT | 4 | 2.00 | 0.75 | - | 0.030 |
| HpT1 | 4 | 2.00 | 0.00 | - | 0.030 |
| HpT2 | 4 | 2.00 | 0.00 | - | 0.030 |
| HpT3 | 4 | 3.00 | 0.00 | - | 0.564 |
